# Supplementary material for: Spatio–temporal modelling of in vitro influenza A virus infection: The impact of defective interfering particles on the type I interferon response
Source: PLoS Comput Biol. 2026 Apr 22;22(4):e1014198. doi: 10.1371/journal.pcbi.1014198 (PMC13128129; doi:10.1371/journal.pcbi.1014198)
Supplement: S1 Appendix — The simulation begins by setting up a hexagonal grid where all cells are initially susceptible. Viruses and DIPs are introduced based on the inoculum size. Infection dynamics unfold through stochastic transitions: susceptible and regrowth cells become infected based on local particle and IFN levels. Infected cells undergo lysis, release particles and IFN, and die (for IAV, virus release occurs by budding rather than lysis, but infected cells ultimately die from infection; the simulation outcome is therefore equivalent). Dead cells regrow if healthy neighbours are present. The model continues until termination criteria are met. Output includes time–resolved data and spatial maps. Animations available at: https://shiny-spatial-infection-app-production.up.railway.app/. Fig B. Dynamics of spread under varying particle–jump behaviours and IFN conditions. Outcomes are shown for scenarios with both DIPs and viruses (top three rows) and viruses only (bottom three rows), under three movement modes: cell–to–cell (left column), finite–radius jumps (middle column), and random jumps (right column). Each row corresponds to a different IFN mode: no IFN, finite–range IFN spread, and global/instantaneous IFN spread. Left plot: temporal dynamics on a log10 scale showing the percentage of cells in each state and global IFN concentration per cell. Right plot: the final spatial distribution of states. Fig C. Heterogeneity in infection dynamics and plaque morphology under varying proportions of cell–to–cell and free–jump spread in Vero cells. Left panels: line plots illustrating the percentage of infected cells over time. Right panels: final plaque morphology at the simulation endpoint (t = 300 hours). Bottom panels: plaque development over time at t = 50, 100, 150, and 200 hours. Fig D. Comparison of plaque dynamics under global IFN with 1% free–jump transmission, in the absence (left) and presence (right) of DIPs. Top panels: time courses showing percentages of antiviral cells, virus–i [file pcbi.1014198.s001.pdf]

S1 Appendix

Supporting Information for

Spatio–temporal modelling of *in vitro* influenza A virus infection: the impact of defective interfering particles on the type I interferon response

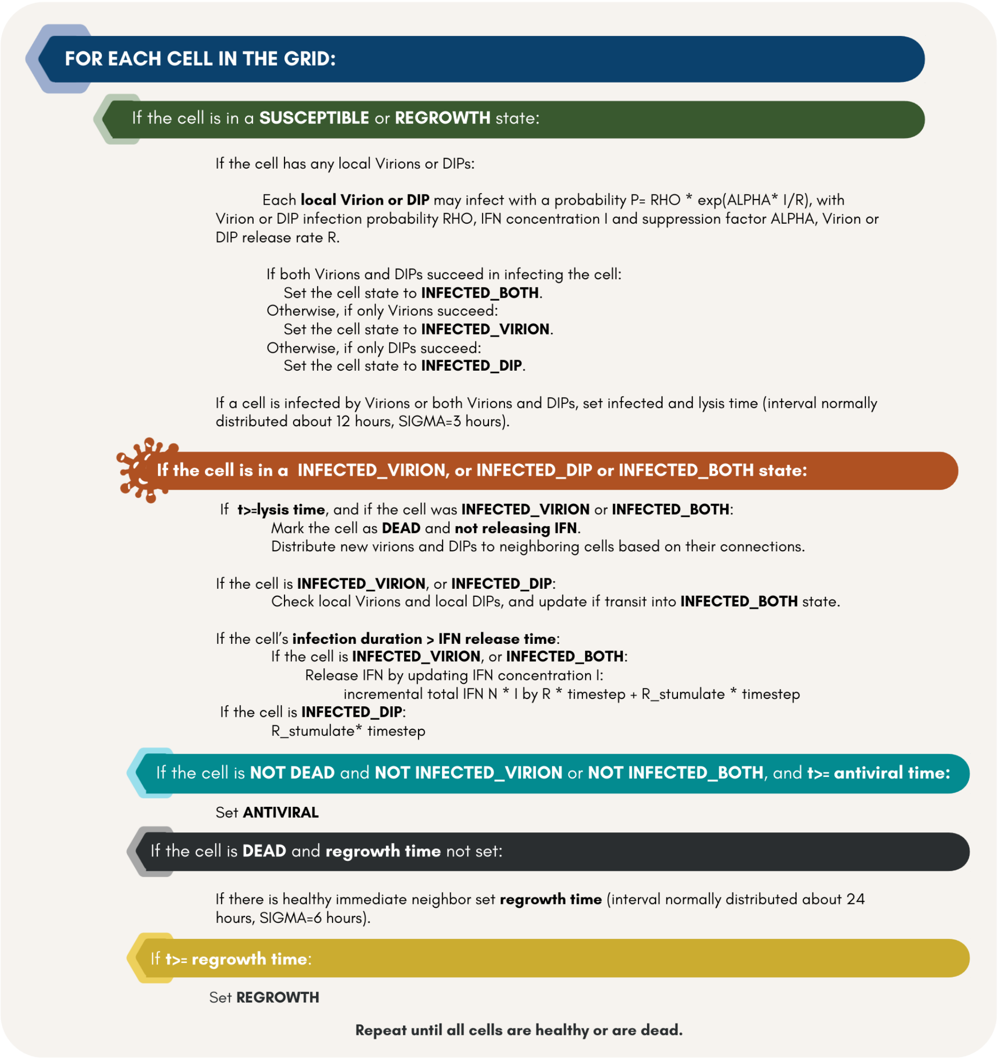

Fig A. Model execution flowchart and explicit transition rules. The simulation begins by setting up a hexagonal grid where all cells are initially susceptible. Viruses and DIPs are introduced based on the inoculum size. Infection dynamics unfold through stochastic transitions: susceptible and regrowth cells become infected based on local particle and IFN levels. Infected cells undergo lysis, release particles and IFN, and die (for IAV, virus release occurs by budding rather than lysis, but infected cells ultimately die from infection; the simulation outcome is therefore equivalent). Dead cells regrow if healthy neighbours are present. The model continues until termination criteria are met. Output includes time–resolved data and spatial maps. Animations available at: `https://shiny-spatial-infection-app-production.up.railway.app/`.

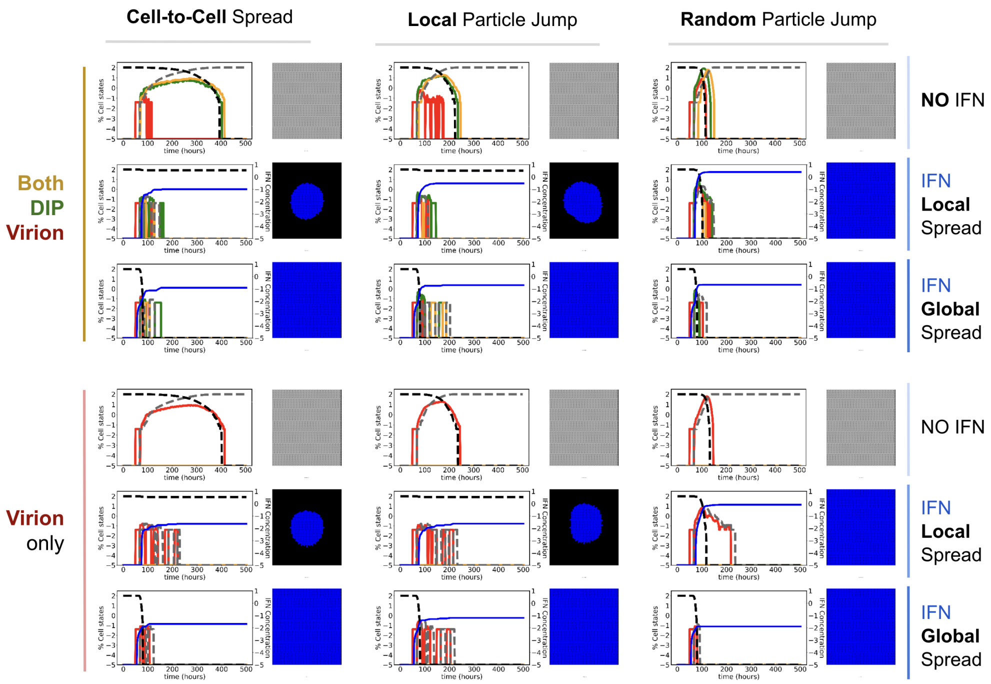

Fig B. Dynamics of spread under varying particle–jump behaviours and IFN conditions. Outcomes are shown for scenarios with both DIPs and viruses (top three rows) and viruses only (bottom three rows), under three movement modes: cell–to–cell (left column), finite–radius jumps (middle column), and random jumps (right column). Each row corresponds to a different IFN mode: no IFN, finite–range IFN spread, and global/instantaneous IFN spread. **Left plot**: temporal dynamics. Both y–axes use a $\log_{10}$ scale: the left y–axis shows the percentage of cells in each state—**virus–only infected**, **DIP–only infected**, **co–infected**, **regrowth**, and **dead**; the right y–axis shows the **global IFN concentration** per cell. **Right plot**: the final spatial distribution of states: **virus–only**, **DIP–only**, **co–infected**, **antiviral**, **susceptible**, and **dead**.

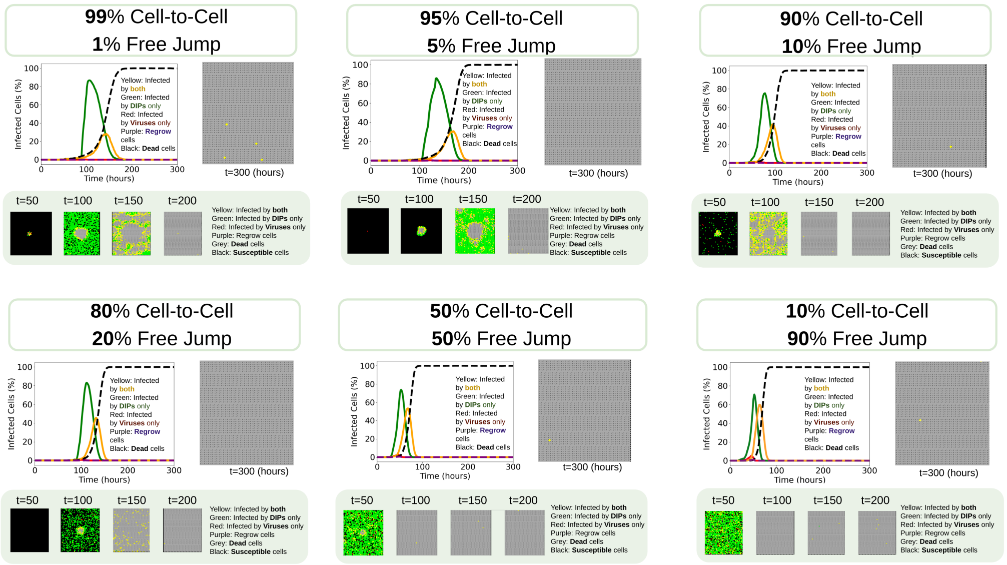

Fig C. Heterogeneity in infection dynamics and plaque morphology under varying proportions of cell–to–cell and free–jump spread in Vero cells. Left panels: The line plots illustrate the percentage of infected cells over time, including viruses–only infections (red), DIPs–only infections (green), coinfections by both (yellow), regrowth cells (purple), and the dashed line represents dead cells (black). Right panels: The final plaque morphology at the simulation endpoint (t = 300 hours), highlighting the spatial distribution of cell states. At this time point, the plaques (grey) have nearly reached full coverage. Bottom panels: Plaque development over time at t = 50, 100, 150, and 200 hours, showing the progression of infection. Colours in the right and bottom panels represent different cell states: infected by both DIPs and viruses (yellow), infected by DIPs only (green), infected by viruses only (red), antiviral state (blue), dead cells (grey), uninfected/susceptible cells (black), and regrowth cells (purple).

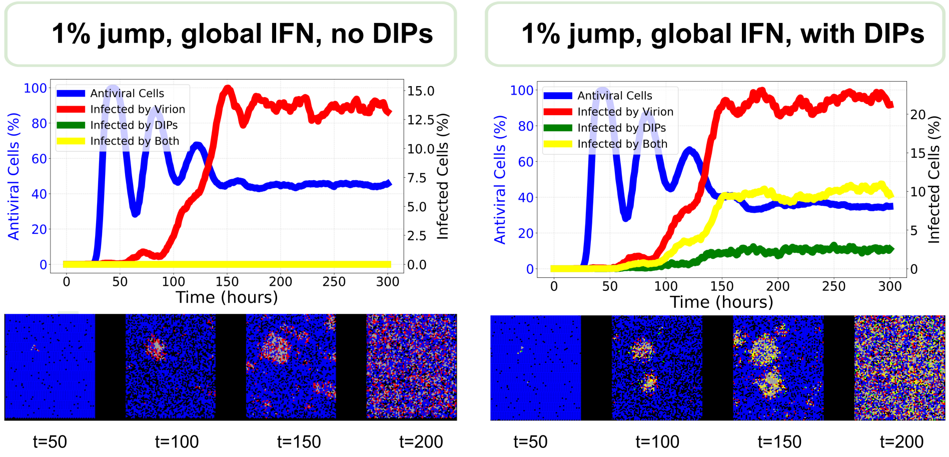

Fig D. Comparison of plaque dynamics under global IFN with 1% free–jump transmission, in the absence (left) and presence (right) of DIPs. Top panels: time courses showing percentages of antiviral cells (blue, left y–axis), virus–infected cells (red), DIP–infected cells (green), and co–infected cells (yellow) (all right y–axis). Bottom panels: representative spatial snapshots at $t = 50, 100, 150,$ and 200 hours, illustrating how DIPs promote co–infection (yellow) and more diffuse plaque morphology despite similar levels of antiviral activation. Colour code: black, susceptible/uninfected; grey, dead; red, virus–infected; green, DIP–infected; yellow, co–infected; blue, antiviral.

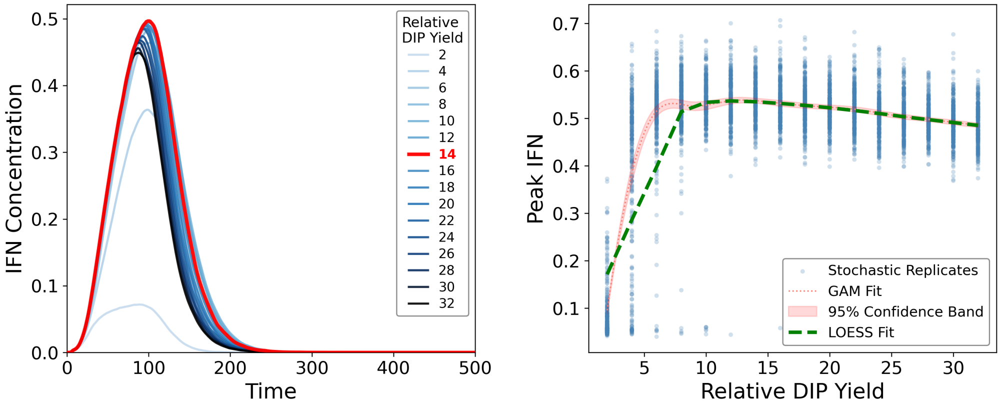

Fig E. IFN dynamics across values of the relative DIP yield under continuous Gaussian release of progeny particles. Left: IFN time series for increasing relative DIP yield. Replacing single-event release with continuous Gaussian release over the intracellular infection period did not remove the non-monotonic response. The highest IFN peak still occurred at an intermediate relative DIP yield, highlighted in red. Right: peak IFN as a function of relative DIP yield across stochastic replicates. Blue points show individual simulations. The GAM fit with its 95% confidence band and the LOESS fit both recovered an interior maximum. Simulation times reflect plaque expansion in an idealized *in vitro* epithelial monolayer rather than the duration of an acute infection in a host.

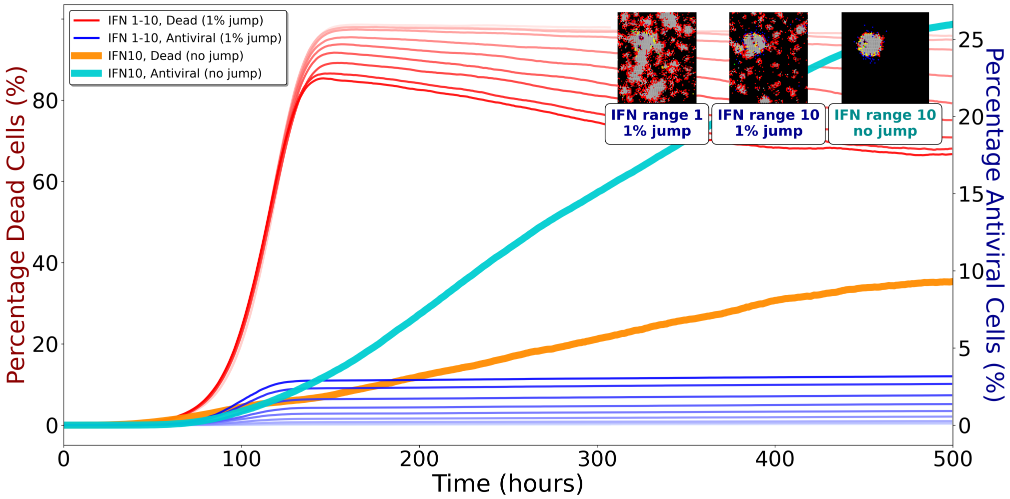

Fig F. IFN range and spreading mode jointly shape plaque dynamics under continuous Gaussian release of progeny particles. Thin red and thin blue curves denote simulations with 1% free-jump transmission for both virions and DIPs, with the remaining 99% spreading by cell-to-cell transmission. Red curves show the percentage of dead cells, and blue curves show the percentage of antiviral cells, across IFN ranges 1–10. Thick orange and thick cyan curves show the corresponding IFN range 10 no-jump controls. As in the burst-release model, even a small fraction of free jumps led to earlier accumulation of dead cells despite stronger antiviral activation, showing that long-range dispersal could still escape local IFN control when particle release was distributed continuously through time. The insets show representative spatial patterns for IFN range 1 with 1% free jumps, IFN range 10 with 1% free jumps, and IFN range 10 with no jumps.

Table A. Parameters corresponding to Figs 2 and 3.

Parameter	Meaning	Value
`TIME_STEPS`	Total simulated duration	25 h
`TIME_STEP`	Simulation time step	1 h
`LysisTime`	Time from infection to lysis (V or V + DIP)	2.0 to 8.0 h
`REGROWTH_MEAN`	Mean time for dead cells to regrow	24.0 h
`REGROWTH_STD`	s.d. of regrowth time	6.0 h
`burstSizeV`	Virions released per lysed cell	800 virions cell^{-1}
`burstSizeD`	DIPs released per lysed cell	900 DIPs cell^{-1}
`RHO`	Per-particle infection rate in $P = \rho \exp(-\alpha I/R)$	0.018 particle^{-1} h^{-1}
`ALPHA`	IFN inhibition strength α	1.0
`R`	IFN scaling factor R	15
`IFN_DELAY`	Mean delay to IFN secretion	5.0 h
`STD_IFN_DELAY`	s.d. of IFN delay	1.0 h
`ifn_half_life`	IFN half-life in medium	4.0 h
`TAU`	Duration of antiviral protection	12.0 h
`virion_half_life`	Half-life of extracellular virions	3.2 h
`DIP_half_life`	Half-life of extracellular DIPs	3.2 h

Table B. Parameters corresponding to Fig 4.

Parameter	Meaning	Value
`TIME_STEPS`	Total simulated duration	145 h
`TIME_STEP`	Simulation time step	1 h
`MEAN_LYSIS_TIME`	Mean time from infection to lysis (V or V + DIP)	12.0 h
`STANDARD_LYSIS_TIME`	s.d. of lysis time (normal distribution)	3.0 h
`REGROWTH_MEAN`	Mean time for dead cells to regrow	24.0 h
`REGROWTH_STD`	s.d. of regrowth time	6.0 h
`burstSizeV`	Virions released per lysed cell	100 virions cell^{-1}
`burstSizeD`	DIPs released per lysed cell	50 DIPs cell^{-1}
`RHO`	Per-particle infection rate in $P = \rho \exp(-\alpha I/R)$	0.015 particle^{-1} h^{-1}
`ALPHA`	IFN inhibition strength α	1.5
`R`	IFN scaling factor R	1
`IFN_DELAY`	Mean delay to IFN secretion	5.0 h
`STD_IFN_DELAY`	s.d. of IFN delay	1.0 h
`TAU`	Duration of antiviral protection	95.0 h
`V_PFU_INITIAL`	Initial virion inoculum	500 PFU
`D_PFU_INITIAL`	Initial DIP inoculum	0 PFU

Table C. Parameters for the base model simulation.

Parameter	Meaning	Value
TIME_STEPS	Total simulated duration	500 h
TIMESTEP	Simulation time step	1 h
MEAN_LYSIS_TIME	Mean time from infection to lysis (V or V + DIP)	12.0 h
STANDARD_LYSIS_TIME	s.d. of lysis time for virion/both infected cells	3.0 h
MEAN_DVG_RECOVERY_TIME	Mean recovery time for DIP–only infected cells (no lysis)	3.0 h
STANDARD_DVG_RECOVERY_TIME	s.d. of DIP–only recovery time	1.0 h
REGROWTH_MEAN	Mean time for dead cells to regrow	24.0 h
REGROWTH_STD	s.d. of regrowth time	6.0 h
burstSizeV	Virions released per lysed cell	50 virions cell^{-1}
burstSizeD	DIPs released per lysed cell	100 DIPs cell^{-1}
RHO	Per-particle infection rate	0.026 particle^{-1} h^{-1}
ALPHA	IFN inhibition strength α	1.0
R	IFN scaling factor R	1
IFN_DELAY	Mean delay to IFN secretion	5 h
STD_IFN_DELAY	s.d. of IFN delay	1 h
ifn_half_life	IFN half-life in medium	4.0 h
TAU	Duration of antiviral protection	12.0 h
virion_half_life	Half-life of extracellular virions	3.2 h
dip_half_life	Half-life of extracellular DIPs	3.2 h
D_only_IFN_stimulate_ratio	Relative IFN production by DIP–only infected cells	5.0 (fold)
BOTH_IFN_stimulate_ratio	Relative IFN production by co–infected cells	10.0 (fold)
V_PFU_INITIAL	Initial virion inoculum	1.0 PFU
D_PFU_INITIAL	Initial DIP inoculum	0.0 PFU
